# Storage stability of texture, organoleptic, and biological properties of goat milk yogurt fermented with probiotic bacteria

**DOI:** 10.3389/fnut.2022.1093654

**Published:** 2023-01-09

**Authors:** Yaling Yang, Ruyue Zhang, Fuxin Zhang, Bini Wang, Yufang Liu

**Affiliations:** College of Food Engineering and Nutritional Science, Shaanxi Normal University, Xi’an, China

**Keywords:** goat yogurt, probiotics, texture, organoleptic characteristics, antioxidant activity (AA)

## Abstract

**Introduction:**

Goat milk is an attractive food due to its high nutritional values, easy digestibility and hypoallergenicity, but has an undesirable “goaty” flavor.

**Methods:**

In this study, goat yogurt was fermented with four probiotics, respectively, including *Lactobacillus acidophilus* (GYA), *Bifidobacterium animalis* (GYB), *Lactobacillus casei* (GYC) and *Lactobacillus plantarum* (GYP), and tested for texture, organoleptic, and biological properties during a 4-week storage period at the refrigerated temperature.

**Results:**

All goat yogurt with probiotics showed an increase on titratable acidity and a corresponding downward trend on pH value. Viable counts of *L. acidophilus* and *L. casei* were above 6 log cfu/mL at the end of the storage, which met the minimum standards for viable probiotic bacteria in yogurt specified by the Food and Agriculture Organization of United Nation (FAO). The texture and organoleptic characteristics of fermented goat milk depended on the strain and the storage period. DPPH free radical scavenging rate and ferric reducing antioxidant power activity gradually increased in all goat yogurts during the storage and yogurt with probiotic bacteria showed higher values than those of GY0.

**Discussion:**

Among all probiotic containing goat yogurts, GYC exhibited the desirable characteristics of hardness, adhesiveness, water holding capacity, antioxidant activity during the whole storage. Furthermore, the addition of *L. casei* effectively weakened the goaty flavor and enhanced the overall acceptability. Thus, fermented goat milk with *L. casei* is optional for the development of goat milk product with satisfactory texture properties, pleasant sensory quality and high bioactivity.

## 1. Introduction

According to statistical data from Food and Agriculture Organization of United Nation (FAO), goat milk is the third milk variety in the world, output of which is only lower than cow milk and buffalo milk. Goat milk is rich in proteins, calcium, antioxidants, antibacterial factors, which meet the requirement for the nutrition and health of human (1). In comparison to bovine milk, goat milk has better digestibility and lower allergenicity (2). In addition, goat milk contains a high level of lysozyme that improves infant immunity, and has been considered as extremely important replacement of human breast milk (3). Nevertheless, goat milk has an undesirable “goaty” flavor, which is generally attributed to volatile components such as branched chain fatty acids (4).

Probiotics are described as live microorganisms that, when ingested in appropriate amounts, confer health advantages to the host (5). Probiotic bacteria confer to goat milk higher antioxidant activity, hypotensive effect and regulatory ability of intestinal flora (6). Ejtahed et al. (7) suggested that ingestion of yogurt with probiotic bacteria might contribute to reduce the cardiovascular disease risk in type 2 diabetes patients (7). Probiotics have powerful antioxidant enzyme system, such as SOD, CAT, GSH-Px, etc. Meanwhile, during the metabolic process, probiotics can also produce organic substances with antioxidant effects such as VE, VC, coenzyme Q, cysteine and so on, which could improve the removal rate of free radicals in the body [V. (8)]. Probiotics prevent hypertension mainly by inhibiting ACE activity, regulating intestinal flora, and enhancing antioxidant capacity. Several bioactive peptides that exert ACE-inhibiting activity have been found in fermented yogurt with probiotics (9). Furthermore, probiotics can also improve the dysbiosis of intestinal flora, leading to the intervene in hypertension. In addition to the health benefits for consumers, probiotic bacteria may develop different patterns of texture and organoleptic properties; each bacterial cultures may lead to a specific product. Probiotic bacteria can create various pleasant volatile flavor compounds during fermentation process (10), and the addition of probiotics contributes to improve rheological properties and organoleptic characteristics of goat milk yogurt. In some cases, yogurt with *L. delbrueckii* ssp. *bulgaricus* was assessed to be too acidic by consumers. Thus, probiotic bacteria were chosen to develop preferred flavors, such as *L. acidophilus, Bifidobacterium* and *L. casei* (11).

Yogurt is traditionally fermented by the combination of *L. delbrueckii* ssp. *bulgaricus* and *S. thermophilus*. In this study, four probiotics were used for the fermentation of goat milk yogurt, including *Lactobacillus plantarum*, *Lactobacillus casei*, *Lactobacillus acidophilus*, and *Bifidobacterium animalis* spp. *lactis* to improve their texture, flavor and bioactive activities. Previous studies have shown that these four probiotic bacteria can survive and maintain a certain vitality in human gastrointestinal tract (12). *L. plantarum* is a kind of facultative heterofermentative lactic acid bacteria. Consumption of fermented milk containing *L. plantarum* brings *in vivo* several benefits to the host (13, 14). *Lactobacillus casei, Lactobacillus acidophilus* and *Bifidobacterium animalis* are also widely used for starter cultures to overcome the issue of post-acidification in yogurt during the storage (15). This study aimed to comprehensively evaluate the effect of probiotics supplementation on goat milk yogurt, including biological activity, texture, and sensory characteristics, during the storage at refrigerated temperature using principal component analysis (PCA). The developed novel goat milk yogurt not only weakens the goaty flavor and improves the sensory acceptance, but also has the health benefits of probiotic combined with goat milk. Hence, it would be much favored by consumers and has a good market prospect.

## 2. Materials and methods

### 2.1. Materials

Raw goat milk was purchased from local farmers was used to ferment yogurt. The starter culture YO-MIX 187 was from Danisco DuPont (Dangé-Saint-Romain, France), which consisted of *Streptococcus thermophilus* and *Lactobacillus bulgaricus*. Four different probiotic fermentation starter, *Lactobacillus plantarum*, *Lactobacillus casei*, *Lactobacillus acidophilus*, and *Bifidobacterium animalis* subsp. *Lactis*, were acquired from Beina Culture Collection (Jiangsu province, China). 2,2-Diphenyl-1-picryl-hydrazyl (DPPH), N-hippuryl-His-Leu tetrahydrate (HHL) and 2,4,6-tripyridyl-s-tria-zine (TPTZ) were purchased from Sigma-Aldrich (Steinheim, Germany). All other chemicals were analytically pure and obtained from Xi’an Chemical Co. (Xi’an, China).

### 2.2. Yogurt preparation

Raw goat milk was pasteurized immediately after the purchase, then cooled to 42°C. Prior to the inoculation, the optical density of four probiotic suspensions at 600 nm was adjusted to 1.0 using sterile saline to ensure that the concentration of each strain of bacteria was about 1.0 × 10^8^ CFU/mL. All treated goat milk were randomly divided into five groups: blank control group (GY0) was only inoculated with 3% YO-MIX187 starter culture; GYA group was inoculated with 3% YO-MIX187 starter culture and *Lactobacillus acidophilus*; GYB group was inoculated with 3% YO-MIX187 starter culture and *Bifidobacterium animalis* ssp. *lactis*; GYC group was inoculated with 3% YO-MIX187 starter culture and *Lactobacillus casei*; GYP group was inoculated with 3% YO-MIX187 starter culture and *Lactobacillus plantarum.* The inoculated goat milk was placed at 42°C for 6 h until they were coagulated, and then all samples were refrigerated at 4°C for 28 days. During the storage, goat milk yogurt was analyzed for texture, organoleptic properties and bioactivity at 1, 7, 14, 21, and 28 days. All the experiments were performed in triplicate.

### 2.3. Determination of the titratable acidity and the pH value

Potentiometric titration was used to determinate the titratable acidity of goat yogurt. The concrete operation referred to the Gharibzahedi’s method (16), and results were expressed as the Thorner degree (°T). The pH value was measured by inserting a previously calibrated pH meter by the electrode into 10.0 g goat yogurt samples. All analysis were conducted for three times.

### 2.4. Viable counts of lactic acid bacteria and probiotics

The viable counts of lactic acid bacteria and probiotics in fermented goat yogurt were determined by plate counting on selective medium. Serial dilutions were made for each goat yogurt sample from 10^–1^ to 10^–7^. *L. bulgaricus* was enumerated on Man, Rogosa, and Sharp (MRS) agar after the anaerobic incubation at 37°C for 72 h; *S. thermophiles* was counted on the modified Chalmers (MC) agar after the aerobic incubation at 37°C for 48 h; the selective medium for *L. acidophilus* was MRS agar medium with clindamycin (17); *Bifidobacteria* colonies were enumerated on the MRS medium with aluminum chloride and sodium propionate on top of MRS agar (MRS-LP) after the anaerobic incubation at 37°C for 72 h (18); The selective medium for *Lactobacillus casei* was LC agar, which consisted of bacteriological peptone, yeast extract, Lab Lemco, sodium acetate, casein hydrolysate, tween 80, KH_2_PO_4_, MgSO_4_, and MnSO_4_ (19); the selective medium of *Lactobacillus plantarum* was configured according to the method of Carmen Bujalance [Bujalance et al. (20)]. The final result was expressed by logarithm of colony forming units per milliliter of yogurt.

### 2.5. Texture measurement

Texture characteristics of goat yogurt were measured by a texture analyzer (Stable Micro System, version 3.0, UK) using A/BE-d835 as the probe, and detailed parameters were set according to Feng’s methods (21). The texture properties, including hardness, adhesiveness, springiness, and cohensiveness, were determined by continuous three compression of each sample.

### 2.6. Water holding capacity determination

The water holding capacity of yogurt reflects the water retention ability of its protein gel network. Briefly, the solidified yogurt was put into a centrifuge tube, which weight was defined as M, and centrifuged at 4,000 g for 10 min. The supernatant was discarded, and the sediment was weighted as M_0_. The water holding capacity (%) could be calculated using the following formula:


$$\mathrm{Water holding capacity}\,(\%)=\frac{M_{0}}{M}\times 100\%$$

### 2.7. Evaluation of biological activity

#### 2.7.1. Antioxidant activity

Ten grams goat yogurt samples were mixed with 30 mL 80% methanol (v/v). The mixture was incubated in an ultrasonic instrument for 20 min at 100 W and then centrifuged at 4,000 g for 10 min. Finally, the supernatant was stored at –20°C prior to the evaluation of DPPH free radical scavenging activity and ferric reducing antioxidant power.

##### 2.7.1.1. DPPH free radical scavenging activity

Half milliliter goat yogurt supernatant was mixed with 4.0 mL DPPH in methanol solution (50.0 mg of DPPH in 1 L of methanol). After the vortex oscillation, the mixture was incubated in dark place for 30 min at room temperature. Ultrapure water was used as blank control and the absorbance at 517 nm was measured by spectrophotometer.

##### 2.7.1.2. Ferric reducing antioxidant power activity

The FRAP analysis was performed according to Benzie and Strain (22) with minor modification (22). Half milliliter supernatant of goat yogurt was mixed with 3.0 mL FRAP reagent. Subsequently, the mixture was water-bathed at 37°C for 10 min and then absorbance at 593 nm was measured with ultrapure water as blank by spectrophotometer. Experimental results were calculated as mmol FeSO_4_/g yogurt.

#### 2.7.2. Antihypertensive activity

The ACE inhibitory activity was evaluated by Xie’s method (23) with slight modification. The pH of goat yogurt was adjusted to 3.4–3.6 with 1 M acetic acid and yogurt samples were centrifuged at 8,000 g for 15 min. Subsequentially, the pH of supernatant was altered to 8.3 with 1 M NaOH, and samples were centrifuged at the equal rotational speed for 20 min. One hundred microliter supernatant was mixed with 200 μL HHL solution (6.5 mmol/L, 0.1 mol/L boric acid solution as solvent) and incubated at 37°C for 5 min. ACE (20 μL, 0.1 U/mL) was added to the mixture and incubated for 30 min at 37°C. A total of 0.25 mL HCl (0.1 mol/L) was added to terminate the reaction. One milliliter ethyl acetate was added to the mixture and incubated at 90°C in the oven. After drying for 1 h, the sample was dissolved in 4 mL ultrapure water and the absorbance value at 228 nm was recorded by ultraviolet spectrophotometer. Before the reaction, ACE was inactivated and added to the sample, which was used as blank control. The ACE activity was calculated using the following formula:


$$\mathrm{ACE inhibitory rate}\,(\%)=(A_{b}-A_{a})/(A_{b}-A_{c})$$

Where A_a_ represents the absorbance value of yogurt; Ab represents the absorbance value of ultrapure water instead of goat yogurt; Ac means the absorbance value of blank control.

### 2.8. Sensory evaluation and flavor compound determination

#### 2.8.1. Sensory evaluation

Sensory analysis was carried out on probiotic goat yogurt that had been refrigerated for 14 days. The panelists (5 males and 5 females, aged between 20 and 35) for the sensory evaluation had undergone initial training and were selected because of their habit of consuming yogurt. Criteria for sensory assessment included pure white, syneresis, acidity, aroma, taste fineness, oral viscosity, goaty flavor, sweetness, bitter, and overall acceptability among goat yogurt samples. The detailed evaluation process was essentially the same as the method described previously (24).

#### 2.8.2. Flavor compound determination

Flavor is strongly dependent on the volatile components of the fermented dairy products (25). Acetaldehyde and diacetyl were two important aroma compounds in yogurt, which differed from milk and other fermented dairy products. In this study, acetaldehyde and diacetyl contents were determined to verify the acceptability and preference of yogurt (26).

##### 2.8.1.1. Acetaldehyde content

Determination of acetaldehyde content in fermented goat yogurt by iodine titration. Twenty milliliter goat yogurt and 16% (v/v) trichloroacetic acid solution with the same volume were fully mixed in a centrifuge tube and centrifuged at 4,500 g for 10 min. At the same time, sterile fresh milk was used as a blank control. Two milliliter 1% NaHSO_3_ solution and 10 mL supernatant of goat yogurt were mixed evenly and incubated at room temperature for 1 h. One milliliter 1% starch solution was added and titrated with 0.10 M iodine standard solution until the solution changed to pale blue. Twenty milliliter NaHCO_3_ (1 M) was further mixed to the above solution under the oscillation until the pale blue disappear. The solution was titrated with 0.01 M iodine standard solution and the volume of standard iodine consumed was recorded. Experiments were repeated for three times.


$$\mathrm{Acetaldehyde content}\,(mg/L)=({\text{V}}_{1}-{\text{V}}_{2})\times \text{C}\times 0.022\times {\text{10}}^{5}$$

Where V_1_ represents the volume of iodine standard solution consumed by the titration for goat yogurt (mL); V_2_ represents the volume of iodine standard solution consumed by the titration in blank control (mL); C represents the concentration of iodine standard solution (M).

##### 2.8.1.2. Diacetyl content determination

O-phenylenediamine colorimetry method was used to determine the content of diacetyl. Five milliliter yogurt sample was mixed with 5 mL 16% trichloroacetic acid (v/v) and 0.5 mL 1% o-phenylenediamine solution (v/v). The mixture was incubated at room temperature for 30 min and 2 mL 4 M hydrochloric acid was added to terminate the reaction. Finally, the optical density was measured by ultraviolet spectrophotometer (EasyPlus, Mettler Toledo) at 335 nm using quartz cuvette. Fresh goat milk was used as blank control.

### 2.9. Statistical analysis

All tests were measured for three times independently. One-way analysis of variances (ANOVA) and Duncan’s multiple-range test were carried out in the statistical analysis by using SPSS Statistics 22.0 to analyze the difference. Only *p*-value of < 0.05 was considered as statistical significance between samples.

## 3. Results and discussion

### 3.1. Changes in titratable acidity and pH of probiotic goat yogurt during the storage

**FIGURE 1:**
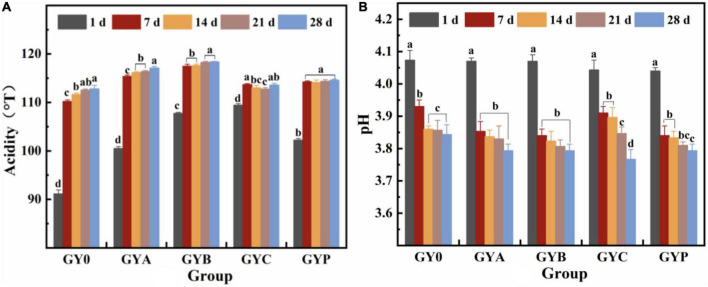
Changes in titratable acidity **(A)** and pH value **(B)** of probiotic goat yogurt stored at 4°C for 1, 7, 14, 21, and 28 days. GY0: goat yogurt fermented with 3% of YO-MIX187 commercial starter culture; GYA: goat yogurt fermented with 3% of YO-MIX187 commercial starter culture and *Lactobacillus acidophilus*. GYB: goat yogurt fermented with 3% of YO-MIX187 commercial starter culture and *Bifidobacterium animalis* ssp. *lactis*, GYC: goat yogurt fermented with 3% of YO-MIX187 commercial starter culture and *Lactobacillus casei* and GYP: goat yogurt fermented with 3% of YO-MIX187 commercial starter culture and *Lactobacillus plantarum*. The meaning of “a–d” refers to the significant differences in acidity and pH of the same experimental group during different storage periods (*p* < 0.05).

The acidity of yogurt is one of the most significant physicochemical indicators of yogurt quality. It is only when the acidity rises to a certain degree that the proteins in goat milk form a gel network and begin to coagulate (27). In addition, acidity plays a key role on the organoleptic characteristics and shelf life of yogurt, thus it is important to monitor the acidity of goat yogurt with probiotic bacteria. Figure 1A visualized the effect of probiotics on the acidity and pH of goat yogurt during the storage at refrigerated temperature. All goat yogurts have an acidity level above 90°T. The titratable acidity of goat yogurt increased with the extension of the storage and pH values presented a corresponding downward trend. Goat yogurt with *Lactobacillus acidophilus* (GYA), *Bifidobacterium animalis* subsp. *Lactis* (GYB) and *Lactobacillus plantarum* (GYP) had more titratable acidity than the sample control (*p* < 0.05). As shown in Figure 1B, there is no significant difference in pH among the experimental groups on the first day of storage. At the end of the storage, the pH values of all goat yogurt with probiotic bacteria were significantly lower than that of the GY0 group (*p* < 0.05). Therefore, the acidification process of goat yogurt is significantly influenced by monoculture of probiotic bacteria used in the manufacturing process. A similar trend was observed by Mituniewicz-Małek et al. (28), who reported a different increase in the titratable acidity of testing milk samples fermented with different probiotic bacteria between day 1 and 21 of storage (28). Bezerra et al. reported the glycolytic effect of added *L. acidophilus*, *L. casei*, *Lactococcus lactis* subsp. *lactis* and *L. paracasei* separately to goat “coalho” cheese and among the bacteria used for supplementation, *L. acidophilus* and *L. paracasei* showed a higher lactose hydrolysis potential with increasing storage time, indicating a consequent increase in acid production that caused a final product with a more abundant flavor and aroma (29).

### 3.2. Changes in the viable counts of lactic acid bacteria and probiotics of goat yogurt during storage

**FIGURE 2:**
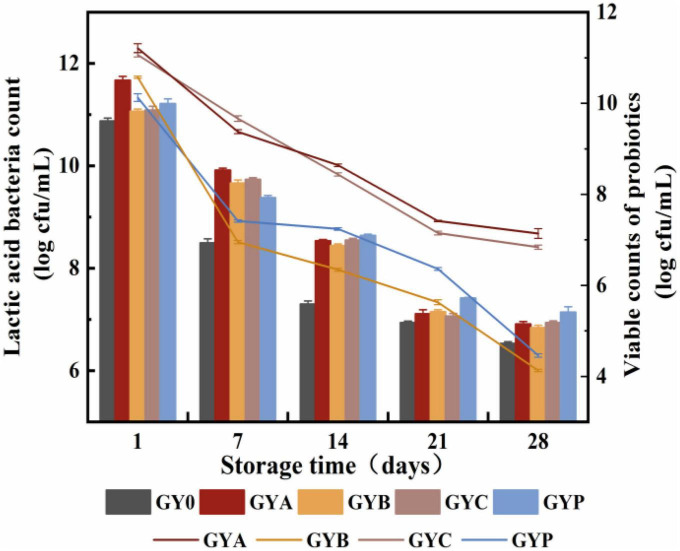
Changes in the viable counts of lactic acid bacteria and probiotics of goat yogurt during the storage.

Differences in the acidity and pH of fermented milk could be correlated with the different production of lactic acid from the growth of lactic acid bacteria. Thus, viable counts of lactic acid bacteria in all goat yogurt were monitored in this study. It could be observed in Figure 2 that the amount of living lactic acid bacteria and probiotic bacteria gradually reduced during the storage of goat yogurt, and GYA and GYC presented more viable counts of probiotic bacteria than the other goat yogurts. It is noteworthy that *L. acidophilus* and *L. casei* in the GYA and GYC group exhibited higher viable counts than 6.0 Log cfu/mL at the end of storage, which met the requirement of the recommended minimum counts of 6.0 Log cfu/mL, showing that goat yogurt could provide a suitable environment for *L. acidophilus* and *L. casei*. *Bifidobacterium animalis* survived at a low level with the prolongation of storage, mainly because the high titratable acidity and production of hydrogen peroxide by *Lactobacillus bulgaricus* (30). Hydrogen peroxide has a certain inhibitory effect on *Bifidobacterium animalis* and reduces its survival rate. Microencapsulation is a process in which the cells are retained within an encapsulating membrane to reduce cell loss or cell injury. Proteins, polysaccharides and prebiotics are frequently used as wall materials for microcapsules to protect and control the release of probiotics (31). Depending on the method used to form the beads, the encapsulation techniques applied to probiotics in fermented dairy products or biomass production can be classified into two groups: emulsion and extrusion. Both emulsion and extrusion techniques can increase the survival of probiotics by 80–95% (32). According to Heidebach et al. (33), casein-based microencapsulation could improve the survival rate of freeze-dried *Bifidobacterium* strains during the storage for up to 90 days. Thus, microencapsulation was a promising way to protect the *Bifidobacterium* strains against the unsuitable environment in goat yogurt.

### 3.3. Effects of added probiotics on textural characteristics of goat yogurt during storage

Texture parameters are considered as an important quality assessment of fermented products that determine whether these products are attractive components in human diet. In the texture test, four indicators directly related to yogurt performance are evaluated: hardness is a parameter that measures the strength of yogurt gel network, and yogurt with higher hardness has better coagulation and more resistance to deformation; adhesiveness reveals its adhesion to the probe, and the thicker yogurt has greater adhesiveness; higher springiness of yogurt leads to a more welcoming taste; cohensiveness reveals the smoothness of goat yogurt, and the smaller cohensiveness, the better smoothness (34). As could be seen from Table 1, the hardness, adhesiveness and cohesion of goat milk yogurt gradually increased during the first 2 weeks, and subsequently decreased with the extension of the storage period, while the springiness showed an opposite tendency. The addition of probiotics had a significant influence on the texture characteristics of goat milk yogurt during the storage period. Several texture characteristics, including hardness and adhesiveness, of goat yogurt were significantly lower than these of cow milk yogurt, which explained that the coagulation of goat milk was worse than cow milk (21).

**TABLE 1:** Changesof texture properties of goat yogurt during the storage.

Texture properties	Group	Storage time (days)				
		1	7	14	21	28
Hardness (g)	GY0	51.418 ± 0.423^a^	53.448 ± 1.309^a^	72.385 ± 1.041^a^	61.073 ± 0.106^a^	57.879 ± 0.533^a^
	GYA	48.533 ± 0.552^b^	52.834 ± 0.300^a^	68.026 ± 1.628^b^	58.445 ± 1.075^b^	53.547 ± 0.375^b^
	GYB	46.352 ± 1.255^c^	52.632 ± 1.509^ab^	61.404 ± 1.039^d^	52.183 ± 0.515^d^	48.935 ± 0.903^c^
	GYC	44.877 ± 0.337^d^	50.827 ± 0.618^b^	69.625 ± 1.352^b^	59.563 ± 0.584^b^	51.894 ± 2.397^b^
	GYP	51.655 ± 0.474^a^	54.169 ± 0.738^a^	63.894 ± 1.052^c^	55.954 ± 1.230^c^	53.410 ± 0.741^b^
Adhesiveness (g⋅s)	GY0	40.010 ± 0.242^b^	61.819 ± 0.452^b^	52.470 ± 1.171^c^	48.599 ± 1.171^b^	42.736 ± 0.886^b^
	GYA	39.409 ± 0.073^b^	49.933 ± 0.797^d^	47.714 ± 0.435^d^	37.457 ± 0.335^c^	35.151 ± 0.287^c^
	GYB	37.277 ± 0.778^c^	44.598 ± 0.477^e^	47.755 ± 0.210^d^	32.396 ± 0.436^d^	26.834 ± 0.303^d^
	GYC	39.678 ± 0.244^b^	63.994 ± 0.675^a^	55.532 ± 0.379^b^	47.572 ± 0.361^b^	42.253 ± 0.344^b^
	GYP	43.253 ± 0.953^a^	56.621 ± 0.505^c^	60.193 ± 0.629^a^	50.501 ± 0.805^a^	44.654 ± 0.409^a^
Springiness	GY0	0.953 ± 0.002^a^	0.947 ± 0.002^bc^	0.929 ± 0.008^b^	0.951 ± 0.003^a^	0.957 ± 0.006^a^
	GYA	0.902 ± 0.009^b^	0.974 ± 0.007^a^	0.948 ± 0.002^a^	0.952 ± 0.001^a^	0.943 ± 0.004^b^
	GYB	0.956 ± 0.005^a^	0.944 ± 0.003^c^	0.935 ± 0.001^b^	0.947 ± 0.001^ab^	0.951 ± 0.004^a^
	GYC	0.953 ± 0.002^a^	0.953 ± 0.004^b^	0.948 ± 0.001^a^	0.945 ± 0.004^b^	0.951 ± 0.002^a^
	GYP	0.947 ± 0.002^a^	0.941 ± 0.002^c^	0.950 ± 0.002^a^	0.945 ± 0.001^b^	0.944 ± 0.003^b^
Cohesion	GY0	26.757 ± 0.203^c^	30.036 ± 0.212^b^	32.591 ± 1.071^b^	28.726 ± 0.403^b^	24.084 ± 0.255^b^
	GYA	27.257 ± 0.122^b^	30.477 ± 0.286^c^	32.790 ± 0.609^b^	26.531 ± 0.438^c^	24.498 ± 0.347^b^
	GYB	17.527 ± 0.392^e^	21.299 ± 0.251^e^	24.739 ± 0.301^c^	24.569 ± 0.528^d^	17.289 ± 0.054^c^
	GYC	28.453 ± 0.183^a^	34.313 ± 0.141^a^	36.180 ± 0.088^a^	30.954 ± 0.271^a^	25.693 ± 0.483^a^
	GYP	24.609 ± 0.095^d^	27.715 ± 0.020^d^	33.532 ± 0.202^b^	26.819 ± 0.142^c^	25.545 ± 0.122^a^

Lowercase letters (a–d) for each indicator in this table indicate significant differences between experimental groups (*p* < 0.05).

**FIGURE 3:**
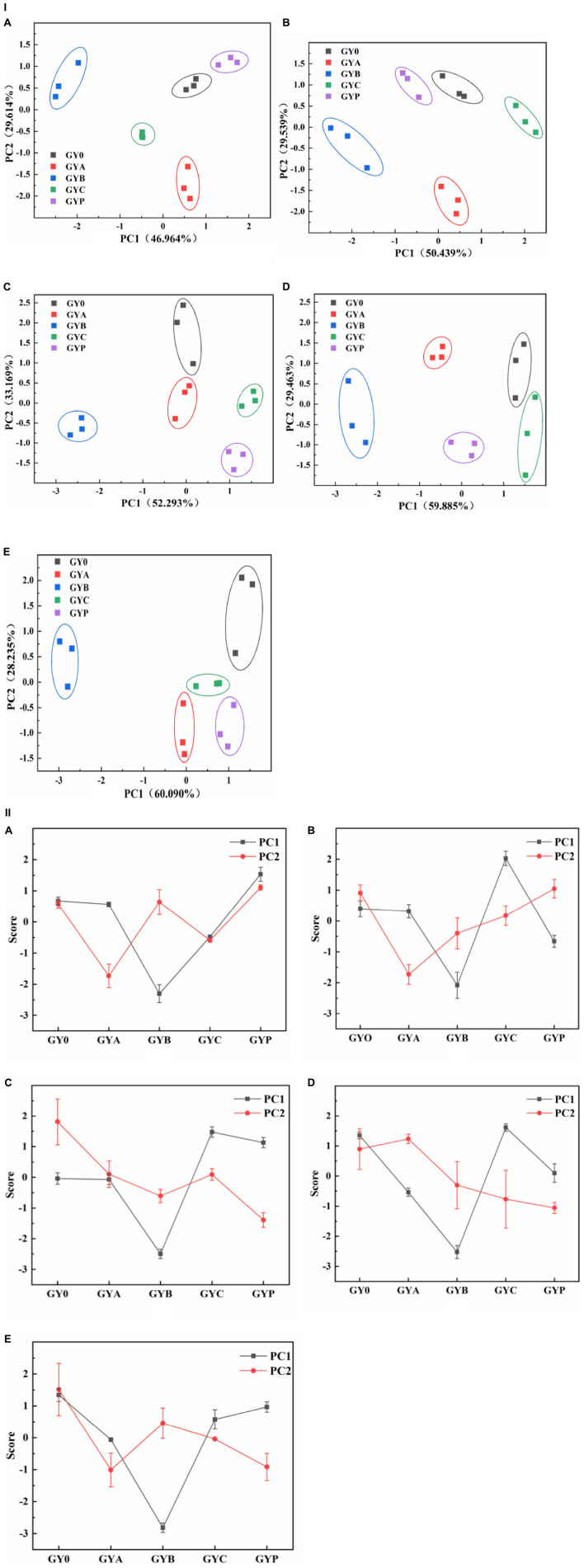
Effect of probiotic supplementation on textural characteristics of goat yogurt during the storage. (I) The principal component analysis scatter plot showed textural properties of goat yogurt at various storage times. (II) The principal component analysis score plot. **(A–E)** Represented the textural principal component analysis of goat yogurt stored for 1, 7, 14, 21, and 28 days, respectively.

PCA is one of the significant statistical techniques for chemometrics and applied to the food quality evaluation. PCA was frequently used to reduce the dimension of the original data by the orthogonal transformation of vector space, which can effectively extract relevant information from complex datasets (35). In this study, the influence of probiotic bacteria on the texture of goat yogurt during the whole storage at the refrigerated temperature was summarized using PCA (Figure 3). During the entire storage of goat yogurt, the cumulative variance contributions of principal component 1 (PC1) and principal component 2 (PC2) are 76.578, 79.978, 85.462, 89.515, and 88.325%, which demonstrates that PC1 and PC2 are representative and well expressed for all indicators of mass structure analysis. Table 2 showed the correlation coefficients between the four qualitative indicators and the PC. When this value is >± 0.7, the PC indicates a strong correlation with each attribute. As shown in Table 2, the indicators correlated with PC1 were hardness, adhesiveness and cohesion, and the indicator correlated with PC2 was springiness. In Figure 3(I), PC1 and PC2 showed a consistent change throughout the whole storage and GYB samples had more distance with GY0 in the PC1 direction than the other probiotic bacteria at the end of the storage, indicating that the addition of *B. animalis* caused the different texture properties of goat yogurt. In Figure 3(II), GY0 obtained the highest scores in both PC1 and PC2 at the end of storage, showing that probiotic bacteria had a negative effect on the texture characteristics of goat yogurt. Numerous reports suggest that the addition of probiotics to dairy products can results in the improvement of textural properties such as hardness, adhesiveness and so on (36). These differences could be attributed to the type of bacterial strains used. The textural characteristics of GYB group were the lowest in all goat yogurt samples in the PC1 direction (Figure 3), probably because the lower pH environment affected the growth and reproduction of *Bifidobacterium animalis* (37).

**TABLE 2:** Factor loading of two principal components during the storage at refrigerated temperature.

Storage (d)	Component	Hardness (g)	Adhesiveness (g⋅s)	Springiness	Cohensiveness
1	1	0.752	0.868	-0.330	0.671
	2	0.431	0.319	0.805	-0.500
7	1	–0.539	0.799	0.383	0.970
	2	0.359	0.591	-0.838	0.043
14	1	0.507	0.765	0.573	0.960
	2	0.846	-0.311	-0.686	0.211
21	1	0.894	0.857	-0.013	0.929
	2	0.364	-0.257	0.985	-0.100
28	1	0.807	0.953	0.070	0.916
	2	0.306	-0.036	0.971	-0.305

### 3.4. Effects of added probiotics on water holding ability of goat yogurt during the storage

**FIGURE 4:**
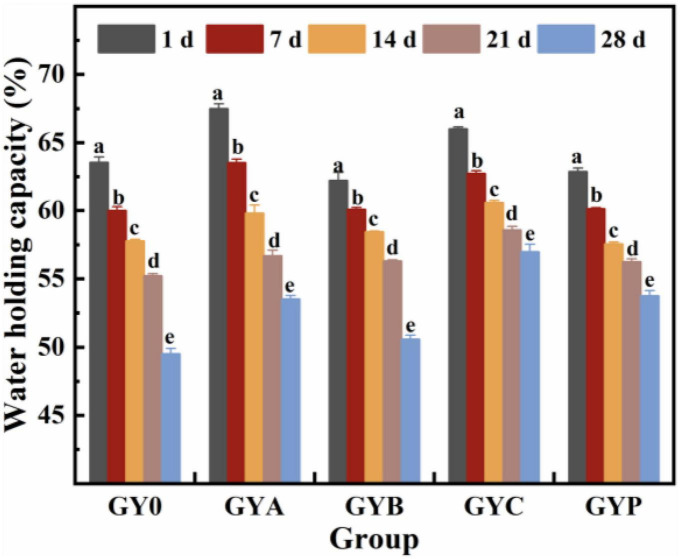
Effect of probiotic supplementation on water holding capacity of goat yogurt during the storage. The meaning of “a–e” refers to the significant differences in water holding capacity of the same experimental group during different storage periods (*p* < 0.05).

Yogurt water holding capacity refers to the ability of yogurt to retain all or part of its own water, and yogurt with low water holding capacity could lead to whey precipitation, thus, resulting in worse quality of fermented milk. As shown in Figure 4, water holding capacity of goat yogurt significantly decreased with the prolongation of storage, and the addition of probiotic bacteria greatly increased the water holding capacities of goat yogurt after 28 days of storage. At the end of storage, the water holding capacity of goat yogurt with probiotic bacteria fluctuated around 53%, indicating that the gel network of probiotic yogurt was relatively dense and compact. This phenomenon could be associated with the secretion of exo-polysaccharide (EPS) by probiotic bacteria, which could interact with proteins in goat milk to enhance the hydration and water holding capacity of yogurt (38). It is worth noting that goat yogurt with L. acidi and L. casei had higher water holding capacity than those for tested yogurt with B. animalis and L. plantarum. It could be speculated that *Lactobacillus acidophilus* and *Lactobacillus casei* might produce more EPS during goat milk fermentation. These results were in concordance with earlier studies reported by Tong Dan et al. (39).

### 3.5. Effects of added probiotics on biological activities of goat yogurt during storage

**FIGURE 5:**
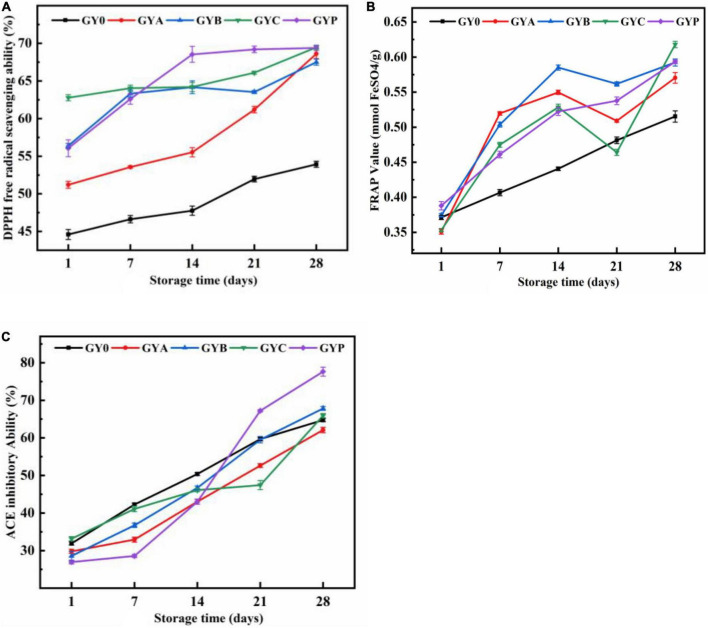
Effect of added probiotics on DPPH free radical scavenging ability **(A)**, ferric reducing antioxidant activity **(B)** and ACE inhibitory activity **(C)** of goat yogurt during the storage.

Figures 5A, B showed the influence of probiotics on the DPPH free radical scavenging capabilities and FRAP values, respectively. DPPH free radical scavenging rate and FRAP value gradually increased in all goat yogurt formulation during the storage and yogurt with probiotic bacteria showed higher values than those of GY0. On the 28th day, the free radical scavenging rate of the four probiotics goat yogurt groups basically reached approximately 70%.

Reactive oxygen species are by-products of natural aerobic metabolisms or hose defense mechanism. Reactive oxygen species-mediated oxidative stress plays a key role in the incidence and development of several chronic diseases, including cancer, diabetes, aging, heart diseases and Alzheimer’s disease. During the last decades, antioxidant substances derived from natural sources have received more and more attention due to their abilities to eliminate reactive oxygen species and radicals. Yogurt has a certain antioxidant effect due to the large amount of amino acids and polypeptides with antioxidant activity produced during the fermentation. Except for the peptides and amino acids, bacterial EPS also have the ability to upregulate the enzymatic and non-enzymatic antioxidant activities by degrading of superoxide anion and hydrogen peroxide. In a previous study from Rahmawati and Suntornsuk (40), compared to yogurt made from ordinary cow’s milk, goat yogurt has considerably better antioxidant activity due to the high protein content of goat milk (40). Probiotic bacteria, as dietary additives, have long history been used to improve the antioxidant activity of foods. *In* both *vivo* and *vitro* experiments, probiotics had antioxidant potential to reduce the oxidative damage to cells. As described by Ejtahed et al. (41), the consumption of probiotic yogurt with *L. acidophilus* and *B. lactis* could improve fasting blood glucose and antioxidant status in type two diabetic patients, thus, resulting in the prevention of diabetes progression (41).

The effect of probiotic bacteria on the antihypertensive activity of goat yogurt was shown in Figure 5C. ACE inhibitory activity of goat yogurt gradually increased with the extension of the storage. Antihypertensive ability of GYP group was higher than those in the other yogurt groups, demonstrating a synergistic effect of *Lactobacillus plantarum* and commercial starter cultures. Antihypertensive function of probiotic yogurt is derived from antihypertensive peptides that inhibit ACE activity during the fermentation, not from the probiotics themselves. Thus, increases in the ACE inhibitory activity during the storage could be explained by the constant hydrolysis of the protein, resulting in the modification of small molecular peptides with ACE inhibition potential (42). The concentration of ACE inhibitory peptides depends on a balance between their formation and degradation into inactive peptide and amino acid (43).

### 3.6. Effect of probiotics on organoleptic evaluation of the yogurt stored for 14 days

**FIGURE 6:**
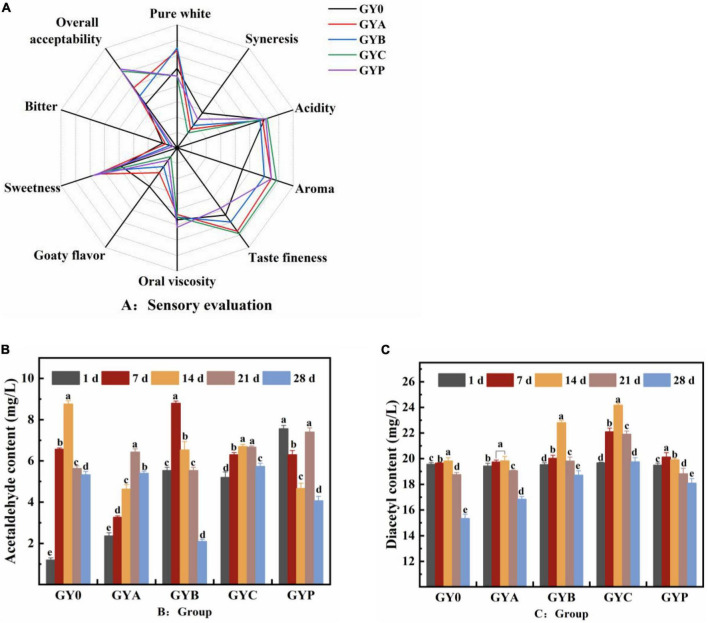
**(A)** The sensory evaluation of probiotic goat yogurt after the storage at the refrigerated temperature for 28 days. **(B)** Changes in acetaldehyde content of probiotic goat yogurt during the storage at 4°C. **(C)** Changes in diacetyl content of goat yogurt fermented with probiotic bacteria during the storage at 4°C. The meaning of “a–e” refers to the significant differences in acetaldehyde content and diacetyl content of the same experimental group during different storage periods (*p* < 0.05).

The organoleptic evaluation of goat yogurt with probiotics was shown in the Figure 6A. It could be seen that the overall acceptability and various indicators of the yogurt containing probiotics were significantly higher than those of the control GY0 group. It showed that the addition of probiotics improved the organoleptic properties of yogurt, especially in terms of flavor and aroma. Moreover, GYC obtained the lowest scores in goaty taste, which indicated that the proper addition of *L. casei* could effectively cover the undesirable goaty aroma in goat milk, thus, improving the flavor of goat yogurt. The butter-like aroma from the fermentation of *Lactobacillus casei* resulted in the highest flavor and aroma scores in GYC group. Several studies pointed out that *Lactobacillus casei* is highly capable of utilizing the proteins in the whey and produces abundant peptides, which was associated with the higher acceptance of GYC group.

The flavor components of goat yogurt are mainly composed of two parts, one is the original volatile compounds in milk, and the other is flavor compounds produced by the fermentation of lactic acid bacteria (26). Acetaldehyde and diacetyl were two important aroma compounds in yogurt, which differ from milk and other fermented dairy products (44, 45). Acetaldehyde is demonstrated to have a green apple or nutty aroma and is considered as the most significant contributor to the typical yogurt flavor. Diacetyl confers a sweet and butter-like aroma and is frequently applied to improve the buttery aroma (46). As is shown in Figure 6B, GY0, GYA, GYB, and GYC groups displayed a significant increase in the acetaldehyde content followed by a decrease at the end of the storage. The acetaldehyde content of both GYB and GYP groups was similar to that of GY0 group. The acetaldehyde content of the GY0 group was always at a high-level during storage, presumably due to the high content of *Streptococcus thermophilus* and *Lactobacillus bulgaricus*. The enzymes secreted by these two bacteria could generate acetaldehyde, and co-cultivation can increase the production of acetaldehyde in goat yogurt (47, 48).

The addition of probiotics had a more significant effect on the diacetyl content in goat yogurt (Figure 6C). The diacetyl concentration of yogurt in the four goat yogurts containing probiotics was greater than that in the GY0 group with the extension of the storage. Especially, the diacetyl concentration in GYC group is above 20 mg/L and significantly higher than those in other groups. These results suggested that the addition of probiotics during the fermentation of goat milk led to the increase of diacetyl, bringing preferred aromatic properties to goat yogurt.

## 4. Conclusion

During the storage, the types of probiotics had a considerable influence on the texture characteristics, microbial viable count and organoleptic of goat yogurt. Moreover, the addition of probiotics could significantly improve the biological activity of goat yogurt, including antioxidant activity and ACE inhibitory activity. GYC presented the best properties of hardness, adhesiveness, water holding capacity, antioxidant activity and overall acceptability. The viable counts in GYC and GYA remained adequate to promote the health benefits to the consumer during the storage at the refrigerated temperature. Based on our results, *L. casei* are recommended for the production of fermented goat milk with satisfactory texture properties, pleasant sensory quality and high bioactivity.

## Data availability statement

The raw data supporting the conclusions of this article will be made available by the authors, without undue reservation.

## Author contributions

YY: investigation and methodology. RZ: writing—original draft. FZ: reviewing and software. BW: conceptualization. YL: supervision and funding acquisition. All authors contributed to the article and approved the submitted version.
